# Supplementary material for: 16p11.2 deletion is associated with hyperactivation of human iPSC-derived dopaminergic neuron networks and is rescued by RHOA inhibition in vitro
Source: Nat Commun. 2021 May 18;12:2897. doi: 10.1038/s41467-021-23113-z (PMC8131375; doi:10.1038/s41467-021-23113-z)
Supplement: Supplementary file 1 — Supplementary Informaion [file 41467_2021_23113_MOESM1_ESM.pdf]

Supplementary Information

16p11.2 deletion is associated with hyperactivation of human iPSC-derived dopaminergic neuron networks and is rescued by RHOA inhibition in vitro

Sundberg M., et al.

Supplementary Fig. 1

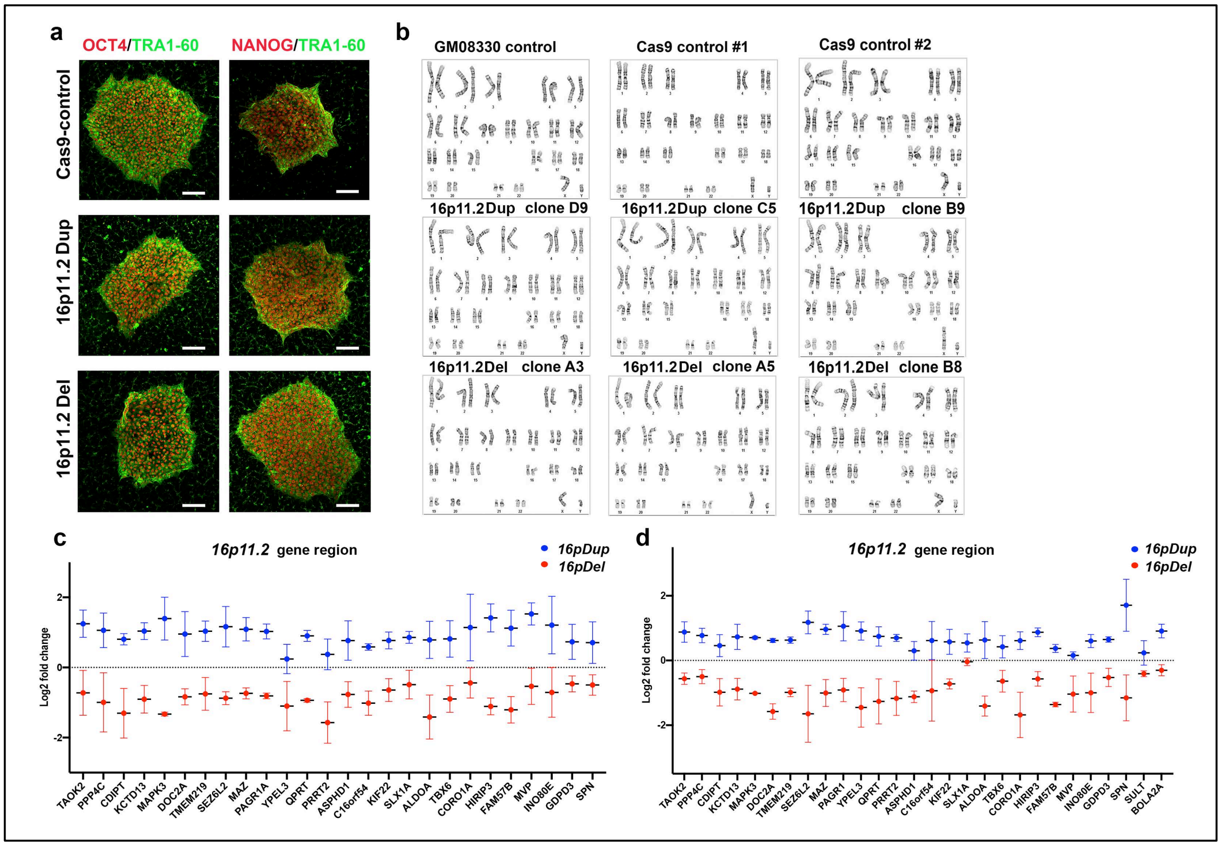

Supplementary Fig. 1 Characterization of 16p11.2 CNV iPSCs and NPCs. a. Human iPSC colonies co-stained with pluripotency markers OCT4/TRA1-60 and NANOG/TRA1-60. Scale bars 82 µm. Experiment was repeated three independent times with similar results. **b.** Karyotypes of the human iPSC lines, from the original control line GM08330 and the CRISPR-clones: cas9-control line, 16pdup and 16pdel. **c.** Expression of the genes in 16p11.2 gene region in the iPSCs, qRT-PCR data is presented as averages ± SEM from three clones per genotype. **d.** Expression of the genes in 16p11.2 gene region in the NPCs at day 18 of differentiation, qRT-PCR data are presented as averages ± SEM from three clones per genotype. Source data are provided as a Source Data file.

Supplementary Fig. 2

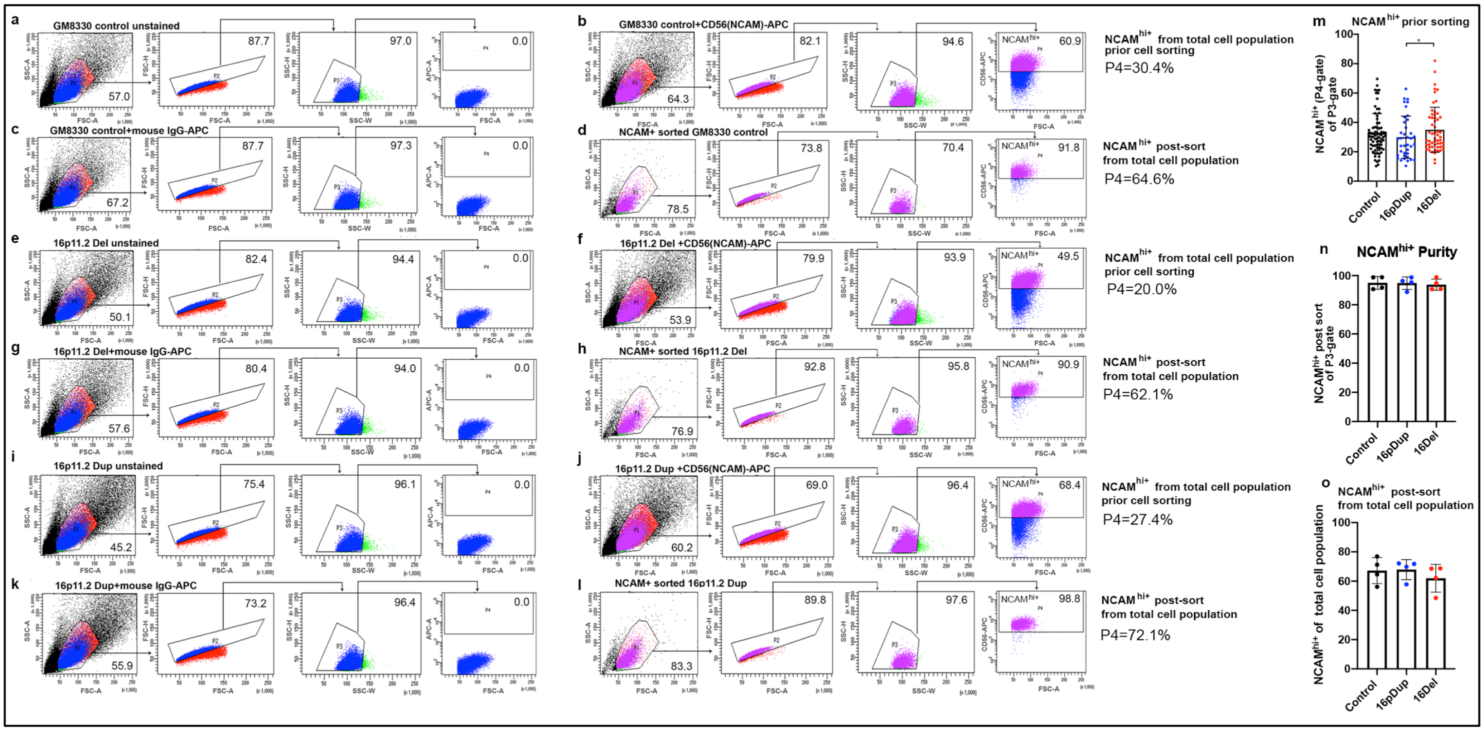

Supplementary Fig. 2 Sorting of iPSC derived NCAM^{hi+} DA neurons. Representative plots of gating cell population of interest and exclusion of the cell debris based on the cell size with side scatter area (SSC-A) and forward scatter area (FSC-A) parameters (P1 gate). P2 gate; gating for exclusion of the doublets with forward scatter height (FSC-H) and FSC-A gate. P3 gate; gating for selection of single cells and exclusion of the doublets with side scatter height (SSC-H) and side scatter width (SSC-W) gate. P4-gate; selection of the cells with highest fluorescent intensity for NCAM^{high+} Allophycocyanin (APC) sorting in dot-plot of Allophycocyanin area (APC-A) (y-axis) and FSC-A (x-axis). P4-gate presents the gate that was used for selection of the cells for sorting and analyses of the percentages of NCAM^{high+} cells from cell population prior cell sorting (data presented in the **Figure 1h**). Gating of unstained cell populations (**a.**, **e.**, **i.**). Gating of NCAM(CD56)-APC (SPM128, Novus Biologicals) stained cells (**b.**, **f.**, **j**.). Gating of cells stained with isotype antibody mouse IgG1-APC (11711, Novus Biologicals) (**c.**, **g.**, **k**.). Gating of sorted NCAM^{high+} cell populations for purity analyses (**d.**, **h.**, **l.**). **m.** Percentage of NCAM^{high+} cells from live single cell population (P3-gate) prior cell sorting, data are presented as mean ± SD. Number of biologically independent samples: n=74 control, n=38 16pDup, n=56 16pDel. Statistical analyses was done with Mann-Whitney test, *$p<0.05$ between 16pDup and 16pDel. **n.** Percentage of NCAM^{high+} purity from P3-gate post-sort, data are presented as mean ± SD, n=4 per genotype. **o.** Percentage of NCAM^{high+} cells post-sort from total cell population, data are presented as mean ± SD, n=4 per genotype.

Supplementary Fig. 3

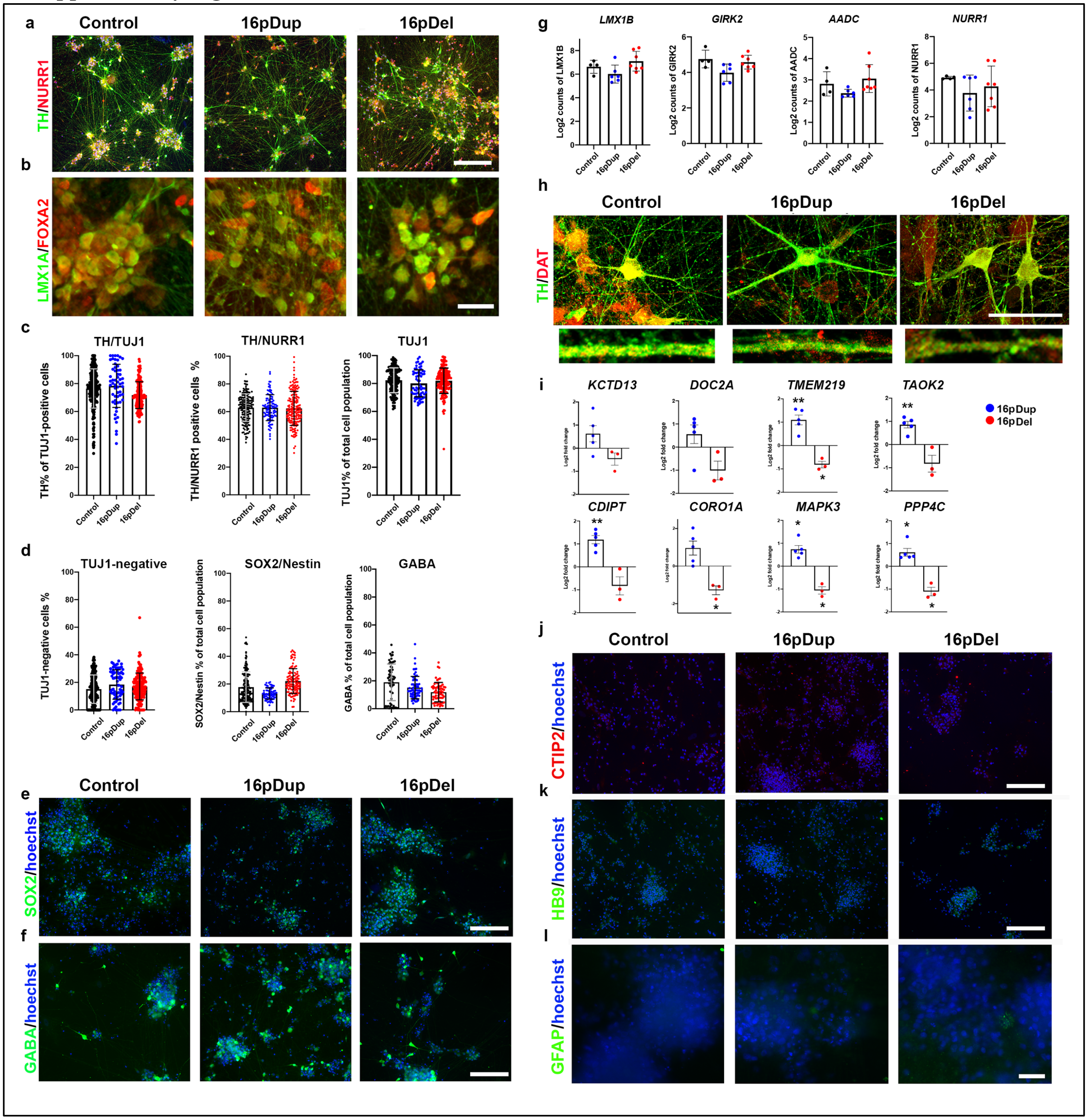

Supplementary Fig. 3 Characterization of iPSC derived DA neurons with 16p11.2 CNVs. a. Representative images of TH/NURR1 double positive cell, scale bar 170 µm, and **b.** FOXA2/LMX1A double positive cells, scale bar 40 µm. Experiment was repeated three independent times with similar results (**a-b**). **c.** Quantification of TH/TUJ, TH/NURR1 and TUJ-positive cells at day 60 of differentiation. **d.** Quantification of TUJ-negative cells, SOX2/Nestin, and GABA positive cells at day 60 of differentiation. Data are presented as mean values ± SD in **c-d**. Number of biologically independent experiments: TH/TUJ1 n=6 control, n=3 16pDup, n=4 16pDel, TH/NURR1 n=4 control, n=3 16pDup, n=4 16pDel, TUJ1 n=6 control, n=3 16pDup, n=5 16pDel, SOX2/Nestin n=4 control, n=3 16pDup, n=3 16pDel, GABA n=7 control, n=7 16pDup, n=7 16pDel. Representative images of SOX2- positive cells (**e.**), and GABA-positive cells in the cell population (**f.**), scale bars 170 µm.

Experiment was repeated three independent times with similar results (**e-f**). **g.** Expression of DA neuron specific genes in the iPSC-derived NCAM^{hi+} sorted DA neurons after ~50 days of differentiation. *LMX1B*, *GIRK2*, *AADC*, *NURR1* were expressed at similar levels between control, 16pdup and 16pdel. Samples were analyzed with the Next-Generation sequencing method (Illumina). Data are presented as mean values ± SD. Number of biologically independent samples n=4 control, n=6 16pDup, n=7 16pDel. **h.** TH-positive neurons express DAT, scale bar 44 μm. Experiment was repeated three independent times with similar results. **i.** QRT-PCR of the selected genes from the 16p11.2 region were analyzed after 56 days of differentiation of the DA neurons. Fold changes are presented in Log2 scale, control value is adjusted to 0, and statistical analyses is calculated compared to control sample (0) with one-sample t test of the mean values of 16pdup and 16pdel samples. *TMEM219* (p=0.0065 16pdup, p=0.0284 16pdel), *TAOK2* (p=0.0038 16pdup), *CDIPT* (p=0.0028 16pdup), *CORO1A* (p=0.0316 16pdel), *MAPK3* (p=0.0117 16pdup, p=0.0220 16pdel), *PPP4C* (p=0.0242 16pdup, p=0.0280 16pdel). Data are presented as mean values ± SEM, p-values *p<0.05, **p<0.01. Number of biologically independent samples n=3 control, n=5 16pDup, and n=3 16pDel. Representative images of CTIP2-negative cells (**j.**), H9B-negative cells **(k.)**, and GFAP-negative cells (**l.**), scale bars 170 μm (**j.-k.**) and 40 μm (**l.**), experiment was repeated two independent times with similar results (**j-l**). Source data are provided as a Source Data file.

Supplementary Fig. 4

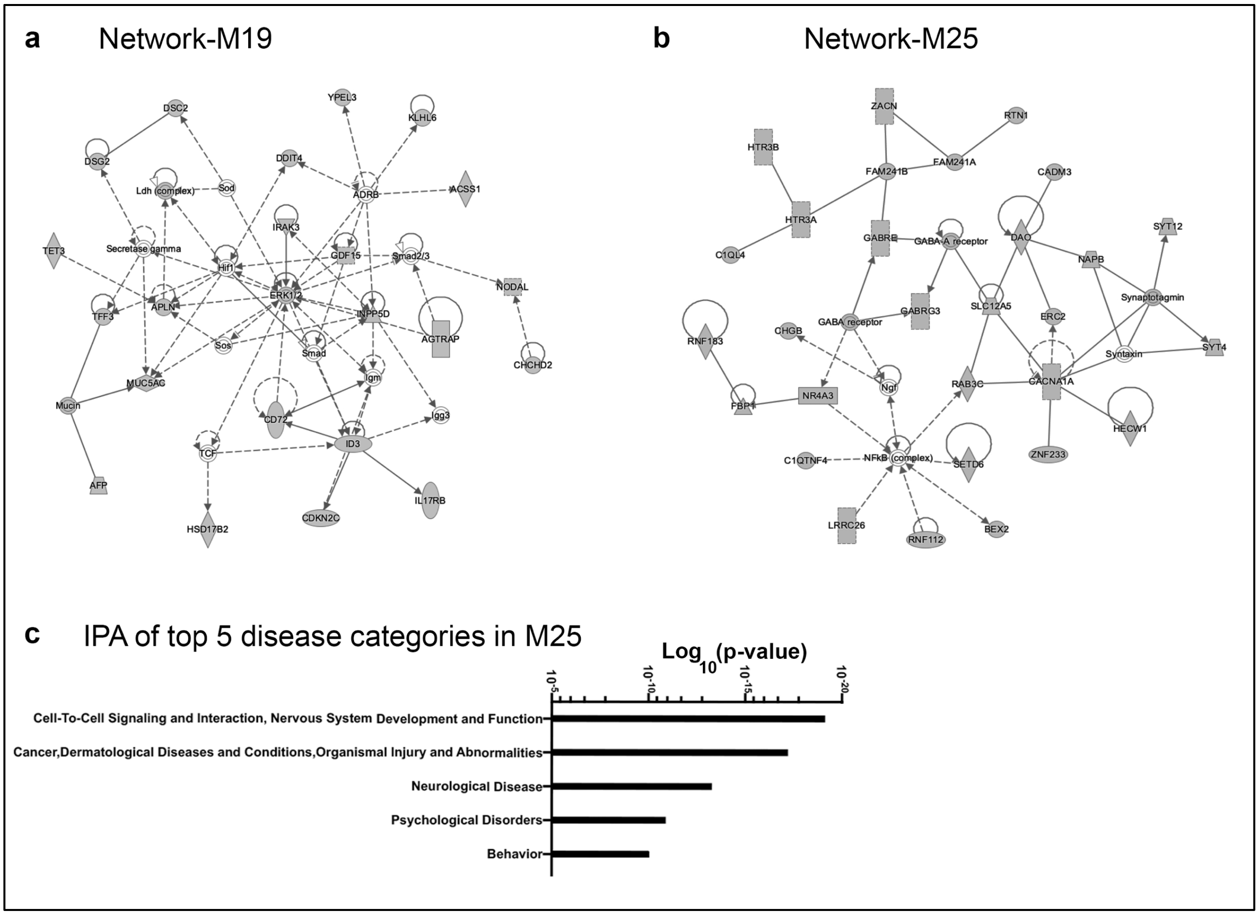

Supplementary Fig. 4 Gene pathway analyses of the genes in the co-expression modules M19 and M25 of 16p11.2 CNV iPSC derived DA neurons. a. Gene network of M19. **b.** Gene network of M25. **c.** Ingenuity pathway analysis of top 5 disease categories of genes in 16pdel DA neurons in M25. Scale $\log_{10}$ (p-value). Ingenuity Pathway Analysis (IPA) (version 57662101) was used for the pathway enrichment analysis in Ingenuity knowledge base (www.qiagen.com/ingenuity). Genes with a kME > 0.5 in either the M19 or the M25 co-expression modules were included in the analysis. Source data of the IPA are provided as a Supplementary Data 3, and 4.

Supplementary Fig. 5

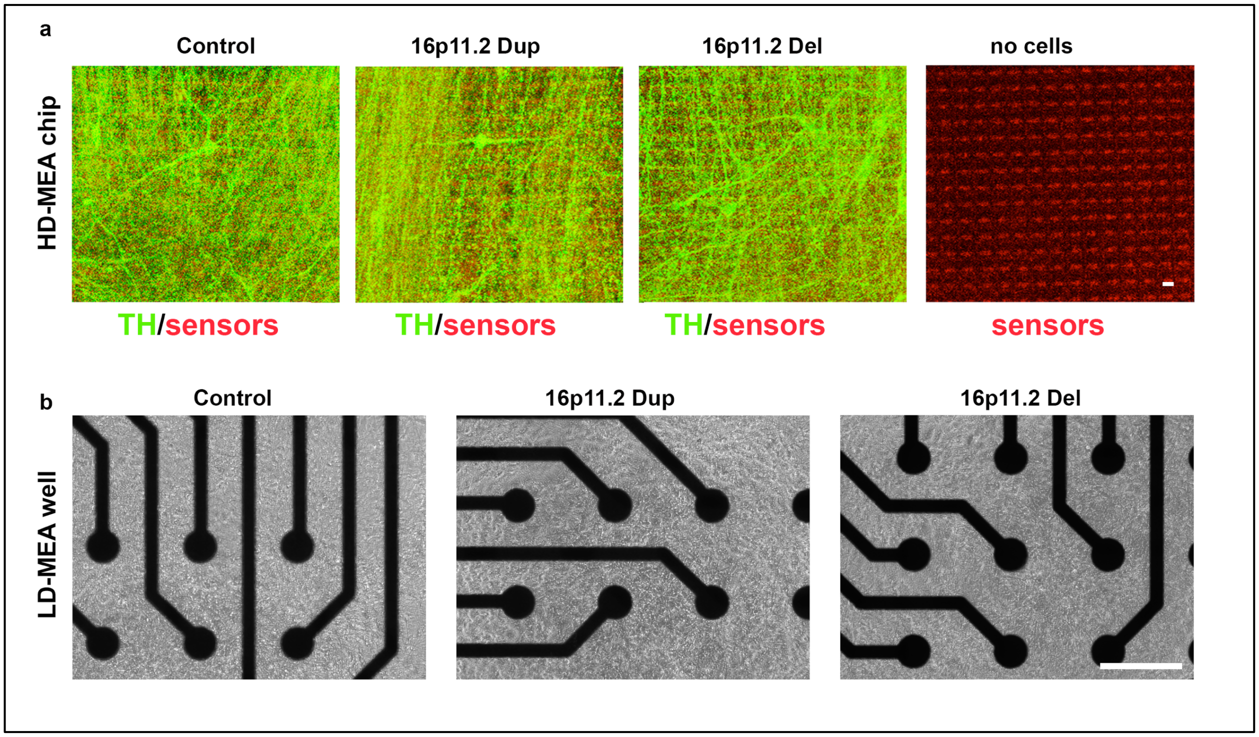

Supplementary Fig. 5 Human iPSC derived DA neurons with 16p11.2 CNVs on HD-MEA and LD-MEA. a. Representative images of iPSC-derived DA neurons that were stained with TH (green) on HD-MEA chips. Sensors are shown in red without cells, one electrode pitch size 17.5 µm. Experiment was repeated three independent times with similar results. **b.** Representative images of the LD-MEA wells of the 48-well MEA-plate show DA neurons plated on top of the electrodes, scale bar 300 µm. Experiment was repeated two independent times with similar results.

Supplementary Fig. 6

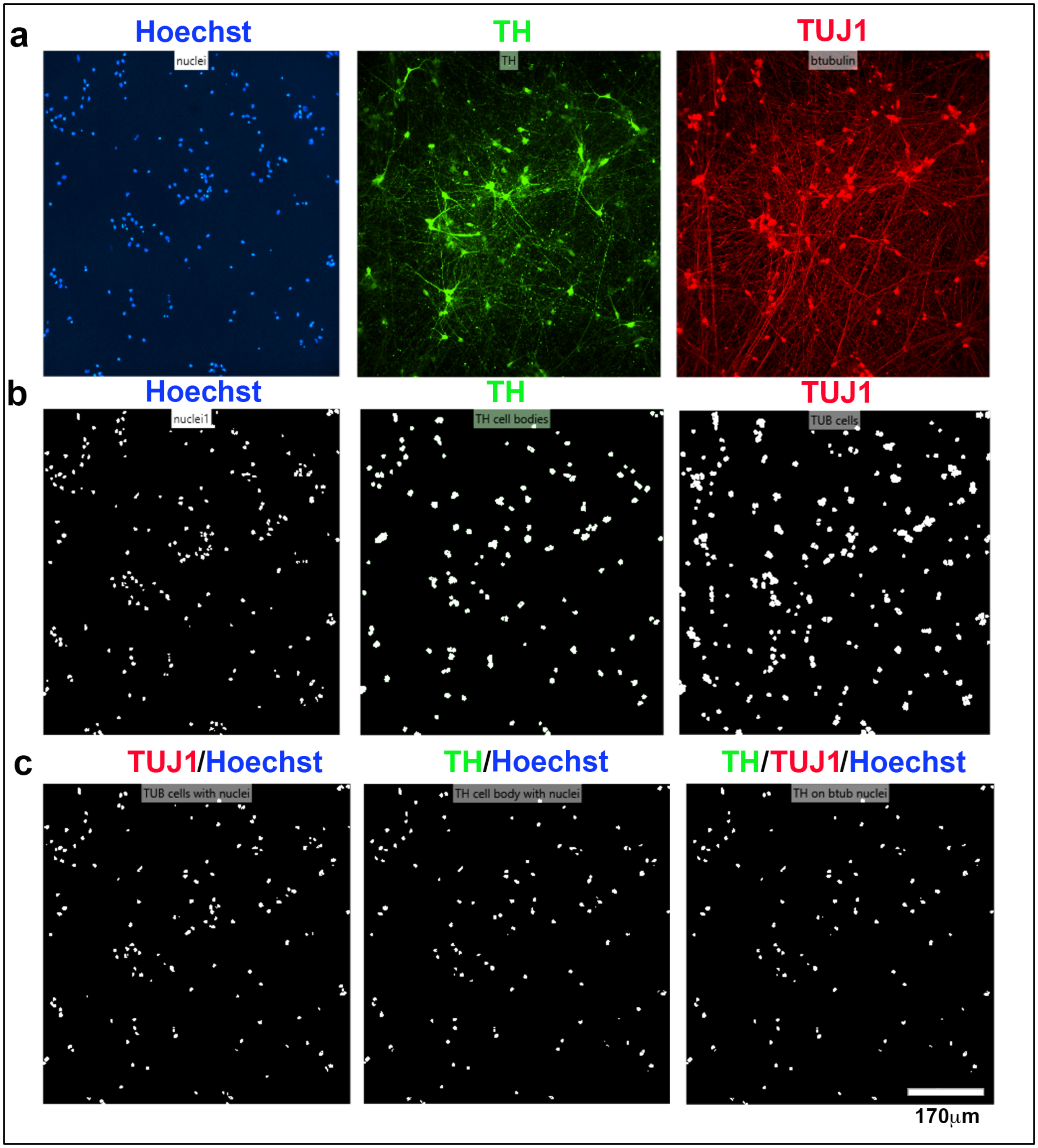

Supplementary Fig. 6 Representative images of the IXM algorithm masks (Molecular Devices) used for quantification of TH/TUJ1 positive neurons in the cell populations. **a.** Panel of Hoechst positive nuclei, TH-positive DA neurons, and TUJ1(b-tubIII) positive neurons. **b.** Representative masks used for quantification of number of nuclei, TH-positive DA-neurons, and TUJ1-positive neurons, and **c.** number of TUJ1/nuclei, TH/nuclei, and TH/TUJ1/nuclei colocalized cells. Scale bar 170 μm. Experiment was repeated three independent times with similar results.

Supplementary Fig. 7

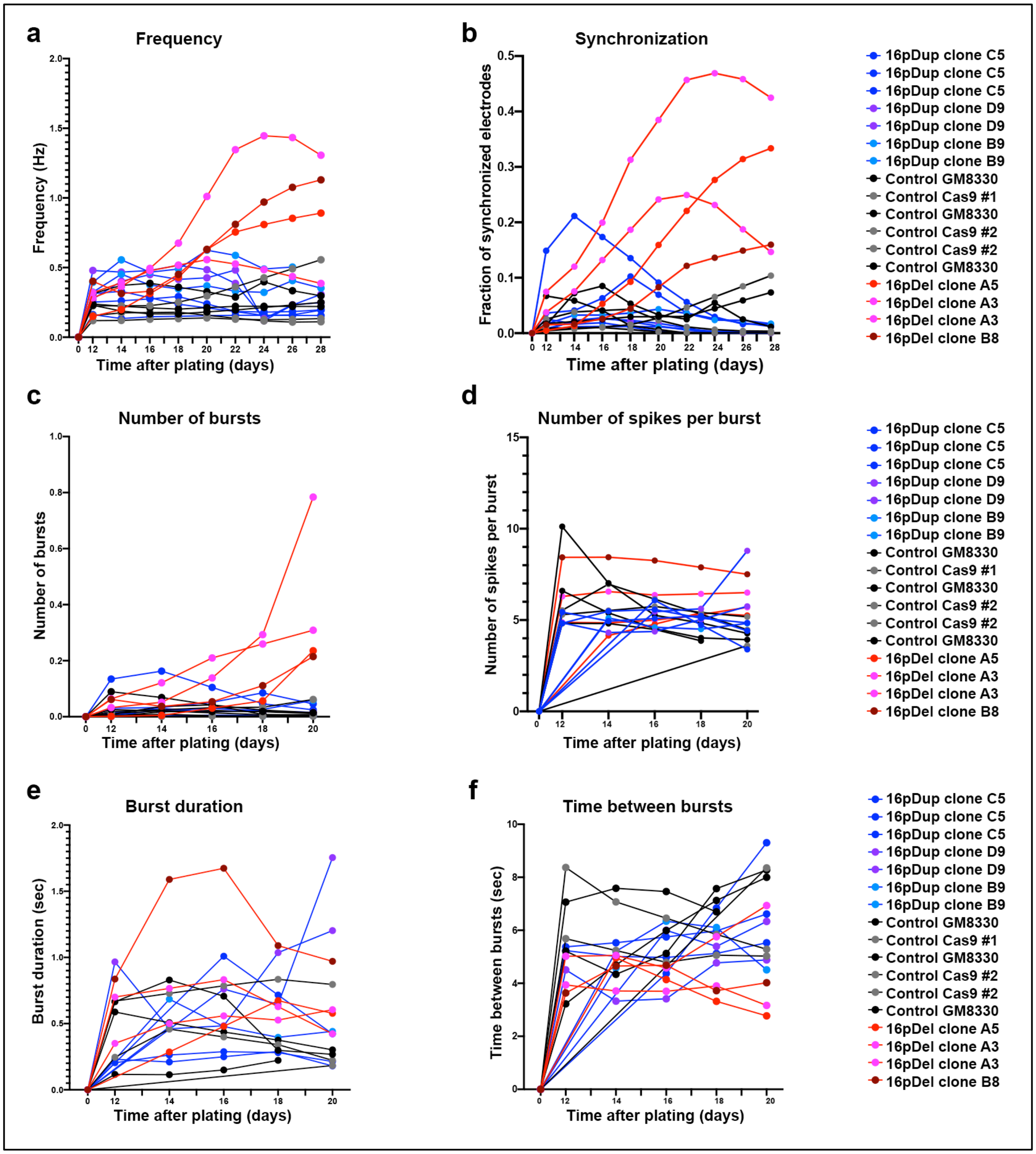

Supplementary Fig. 7 HD-MEA data of individual iPSC-clones used for DA neuron network characterization. **a.** Frequency of active sensors (Hz). **b.** Fraction of synchronized sensors over all sensors. **c.** Number of bursts per minute over all sensors. **d.** Number of spikes per burst. **e.** Burst duration. **f.** Time between bursts. Source data are provided as a Source Data file.

Supplementary Fig. 8

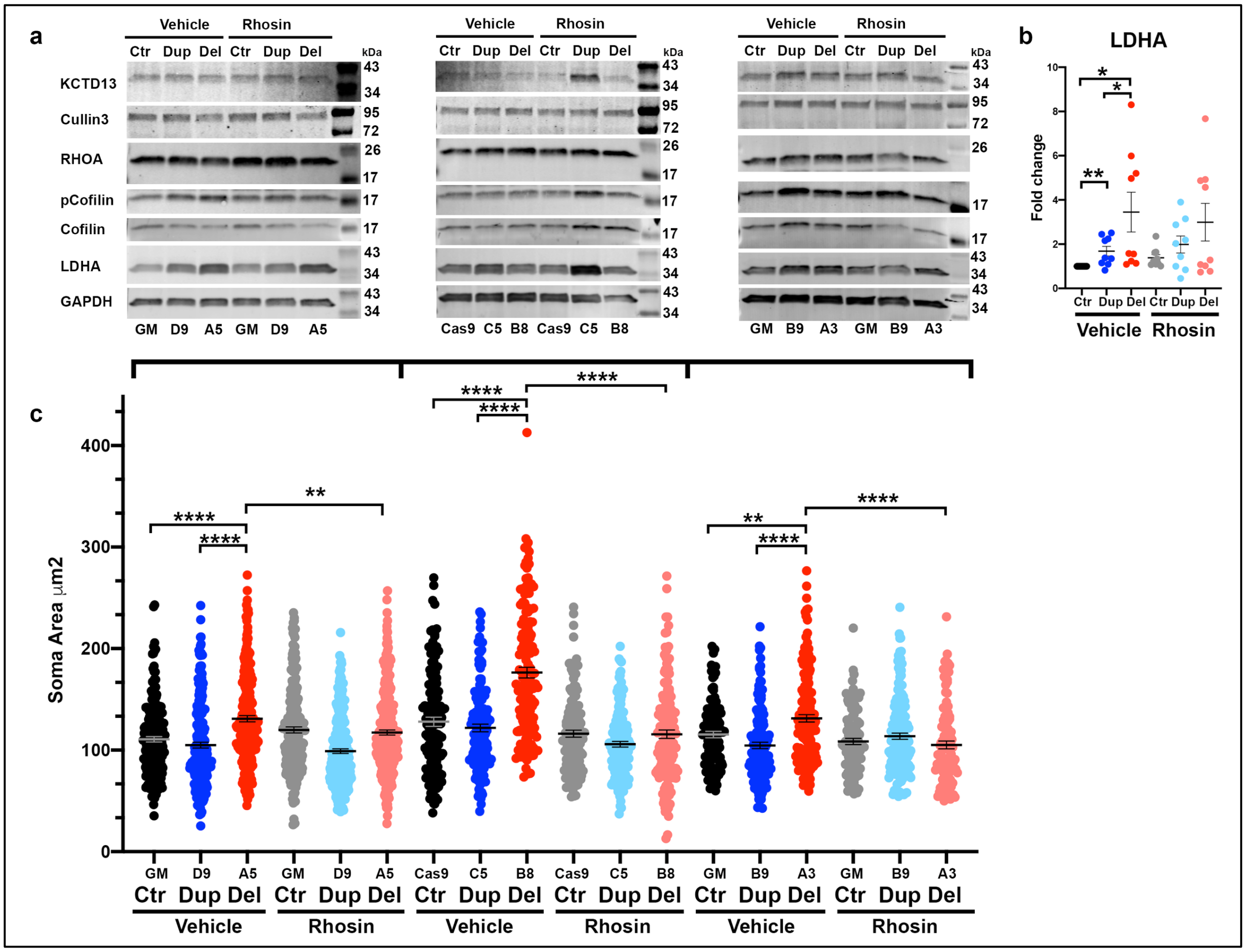

Supplementary Fig. 8 Characterization of KCTD13-Cullin3-RHOA pathway and soma size of iPSC derived DA neurons with 16p11.2 CNVs. a. Representative western blots of KCTD13-Cullin3-RHOA pathway analyses of individual iPSC-clones used for DA neuron differentiation and vehicle or Rhosin treatments. Experiment was repeated three independent times with similar results. **b.** Quantification of LDHA expression intensities show that 16pdel cells had increased expression levels of LDHA compared to control cells (p=0.0131), and compared to 16pdup cells (p=0.0443). 16pdup cells also had increased expression of LDHA compared to control cells (p=0.0069). Unpaired t-test with Welch's correction and one tailed p-values, data are presented as mean values ± SEM, *p<0.05, **p<0.01. Number of biologically independent samples n=9 control, n=9 16pDup, n=9 16pDel. **c.** TH+ cell soma size analyses of individual clones used for DA neuron differentiation and vehicle or Rhosin treatments. One-way ANOVA and Tukey's multiple comparisons tests with adjusted p-values were calculated between samples of each individual batch. Batch1: A5-del vs GM-control (p<0.0001), A5del vs D9dup (p<0.0001), A5del+veh vs A5del+rhosin (p=0.0032). Batch2: B8-del vs Cas9-control (p<0.0001), B8del vs C5dup (p<0.0001), B8del+veh vs B8del+rhosin (p<0.0001). Batch3: A3-del vs GM-control (p=0.0082), A3del vs B9dup (p<0.0001), A3del+veh vs A3del+rhosin (p<0.0001). Data are presented as mean values ± SEM, **p<0.01, ****p<0.0001. Number of biologically independent experiments per genotype n=6 for vehicle and n=6 for Rhosin treated cells. Source data are provided as a Source Data file.

Supplementary Table 1. a. SNP array data of human iPSC lines.

Sample ID	Clone name	Identification	SNP array data (16p11.2 del/dup)	Additional CNVs
Control	GM08330	Untreated control GM08330 iPSC	No del/dup	15kbp gain at 1p21.1 (chr1:104,113,158−104,128,618); 0.328Mbp loss at 4q22.2 (chr4:94,129,862-94,458,391); 123kbp loss at 5q23.3 (chr5:127,742,721−127,866,311); 75kbp gain at 12q13.13 (chr12: 52,702,656-52,777,536);0,933Mb gain (75% mosaic) at 20q11.21 (chr20:29,820,178−30,753,270).
Control	Cas9-control	CRISPR-Cas9 treated control iPSC	No del/dup	48kbp gain at 1p21.1 (chr1:104,108,181−104,155,944); 0.328Mbp loss at 4q22.2 (chr4:94,129,862-94,458,391); 124kbp loss at 5q23.3 (chr5:127,742,721−127,866,311);85kbp gain at 12q13.13 (chr12: 52,702,656-52,788,012);0,933Mb gain (75% mosaic) at 20q11.21 (chr20:29,820,178−30,753,270).
Del	A3 del	CRISPR_Del	0.620Mb del	48kbp gain at 1p21.1 (chr1:104,107,068−104,154,934); 0.328Mb loss at 4q22.2 (chr4:94,129,862-94,458,391); 124kbp loss at 5q23.3 (chr5:127,742,721−127,866,311);85kbp gain at 12q13.3 (chr12:52,702,656-52,788,012); 0,933Mb gain at 20q11.21 (chr20:29,820,178−30,753,270).
Del	A5 del	CRISPR_Del	0.620Mb del	42kbp gain at 1p21.1 (chr1:104,113,158−104,154,934); 0,329Mb loss at 4q22.2 (chr4:94,129,862-94,458,391); 50kbp loss at 5p15.1 (chr15: 15,598,629-15,648,296); 124kbp loss at 5q23.3 (chr5:127,742,721−127,866,311);77kbp gain at 12q13.13 (chr12: 52,702,656-52,779,391); 0,933Mb gain (75% mosaic) at 20q11.21 (chr20:29,820,178−30,753,270).
Del	B8 del	CRISPR_Del	0.620Mb del	42kbp gain at 1p21.1 (chr1:104,113,158−104,154,934); 0,329Mb loss at 4q22.2 (chr4:94,129,862-94,458,391); 124kbp loss at 5q23.3 (chr5:127,742,721−127,866,311); 59kbp gain at 12q13.13 (chr12: 52,702,656-52,761,959); 0,948Mb gain (70% mosaic) at 20q11.21 (chr20:29,805,100−30,753,270).
Dup	B9 dup	CRISPR_Dup	0.620Mb dup (gain 70%)	20kbp gain at 1p21.1 (chr1:104,109,722−104,129,880); 0,329Mb loss at 4q22.2 (chr4:94,129,862-94,458,391); 124kbp loss at 5q23.3 (chr5:127,742,721−127,866,311); 59kbp gain at 12q13.13 (chr12: 52,702,656-52,761,959); 0,933Mb gain (85% mosaic) at 20q11.21 (chr20:29,820,178−30,753,270).

Dup	C5 dup	CRISPR_Dup	0.552Mb dup (gain 60%)	17kbp gain at 1p21.1 (chr1:104,113,158−104,129,880); 0,329Mb loss at 4q22.2 (chr4:94,129,862-94,458,391); 126kbp loss at 5q23.3 (chr5:127,742,721−127,869,190); 75kbp gain at 12q13.13 (chr12: 52,702,656-52,777,536); 100kb gain at 19q13.42 (chr:54,464,398-54,563,875); 0,933Mb gain (75% mosaic) at 20q11.21(chr20:29,820,178−30,753,270).
Dup	D9 dup	CRISPR_Dup	0.784Mb dup (gain 70%)	32kbp gain at 1p21.1 (chr1:104,122,556−104,154,934); 0,329Mb loss at 4q22.2 (chr4:94,129,862-94,458,391); 124kbp loss at 5q23.3 (chr5:127,742,721−127,866,311); 75kbp gain at 12q13.13 (chr12: 52,702,656-52,777,536); 0,948Mb gain (70% mosaic) at 20q11.21 (chr20:29,805,100−30,753,270).

Supplementary Table 1. a. SNP array data of undifferentiated iPSC-lines. The control iPSC lines (GM08330 control and Cas9-treated control line) didn't carry deletions or duplications of the 16p11.2 gene region. Deletion of 16p11.2 region was detected in all the 16pdel clones; A3, A5, and B8. Gain of 16p11.2 was detected in all the 16pdup clones; B9, C5, D9. The naïve iPSC-line (GM08330) and all the CRISPR-cas9 edited iPSC clones harbored a 0.9 Mb gain on chromosome 20; 20q11.21. The ratio shift of this copy number change indicates possible mosaicism of approximately 70%-85%. Gain of 20q11.21 is recurrent change in human pluripotent stem cell cultures [93].

Supplementary Table 1. b. aCGH array data of human iPSC lines.

Sample ID	Clone name	Identification	aCGH array data (16p11.2 del/dup)	Additional CNVs
Control	GM08330	Untreated control GM08330 iPSC	No del/dup	103.5 Kb gain at 1p21.1 (chr1:104107530−104211056); 323.1 Kb loss at 4q22.2 (chr4:94128586−94451650); 116.7 Kb loss at 5q23.3 (chr5:127749595−127866319);1.0 Mb gain at 20q11.21 (chr20:29833609−30855697).
Control	Cas9-control	CRISPR-Cas9 treated control iPSC	No del/dup	103.5 Kb gain at 1p21.1 (chr1:104107530−104211056); 323.1 Kb loss at 4q22.2 (chr4:94128586−94451650); 116.7 Kb loss at 5q23.3 (chr5:127749595−127866319); 1.0 Mb gain at 20q11.21 (chr20:29833609−30855697).
740 kb del	A3 del	CRISPR_Del	545.6 kb del	103.5 Kb gain at 1p21.1 (chr1:104107530−104211056); 323.1 Kb loss at 4q22.2 (chr4:94128586−94451650); 116.7 Kb loss at 5q23.3 (chr5:127749595−127866319); 1.0 Mb gain at 20q11.21 (chr20:29833609−30855697).
740 kb del w/SD dup	A5 del	CRISPR_Del	545.6 kb del	103.5 Kb gain at 1p21.1 (chr1:104107530−104211056); 323.1 Kb loss at 4q22.2 (chr4:94128586−94451650); 116.7 Kb loss at 5q23.3 (chr5:127749595−127866319); 1.0 Mb gain at 20q11.21 (chr20:29833609−30855697).
740 kb dup	B9 dup	CRISPR_Dup	544.3 kb dup	103.5 Kb gain at 1p21.1 (chr1:104107530−104211056); 323.1 Kb loss at 4q22.2 (chr4:94128586−94451650); 116.7 Kb loss at 5q23.3 (chr5:127749595−127866319); 1.0 Mb gain at 20q11.21 (chr20:29833609−30855697).
740 kb dup	C5 dup	CRISPR_Dup	679.6 kb dup	103.5 Kb gain at 1p21.1 (chr1:104107530−104211056); 323.1 Kb loss at 4q22.2 (chr4:94128586−94451650); 116.7 Kb loss at 5q23.3 (chr5:127749595−127866319); 1.0 Mb gain at 20q11.21 (chr20:29833609−30855697).
740 kb dup	D9 dup	CRISPR_Dup	544.3 kb dup	103.5 Kb gain at 1p21.1 (chr1:104107530−104211056); 323.1 Kb loss at 4q22.2 (chr4:94128586−94451650); 116.7 Kb loss at 5q23.3 (chr5:127749595−127866319);1.0 Mb gain at 20q11.21 (chr20:29833609−30855697).

Supplementary Table 1. b. The aCGH data (Tai et al, 2016) shows consistent results of the CRISPR-cas9 edited human iPSC lines compared with the SNP array data (Supplementary Table 1.a.).

Supplementary Table 1. c.

Gene	ID number	nr	Control Mean	Control SD	16pDel Mean	16pDel SD	fold change Ctr vs Del	pval	FDR
BCL2L1	ENST00000376062	TSS83649	6.79550772	0.24385635	6.71701433	0.20562474	-0.078493	0.597248612	0.96123098

Gene	ID number	nr	Control Mean	Control SD	16pDup Mean	16pDup SD	fold change Ctr vs Dup	pval	FDR
BCL2L1	ENST00000376062	TSS83649	6.79550772	0.24385635	6.5519989	0.26902017	-0.243508	0.12278779	0.49729806

Gene	Control Mean	SD	N	16pDup Mean	16pDup SD	N	16pDel Mean	16pDel SD	N
BCL2L1	6.79550772	0.24385635	4	6.5519989	0.26902017	6	6.71701433	0.20562474	7

Supplementary Table 1. c. RNAseq analyses detected that there were no significant differences in the expression levels of *BCL2L1* between different genotypes of the iPSC-derived DA neurons. We performed the initial differential gene expression analysis with LIMMA (Linear models for microarray and RNAseq data, R/Bioconductor software package). The statistical analyses was done with modified Fisher's Exact Test p-value, one sided, and correction for multiple comparisons was made with the FDR. For comparison of the *BCL2L1* expression between different genotypes we did a statistical test for the individual gene expression values of each sample group with one-way Brown-Forsythe and Welch ANOVA, followed by Dunnett's T3 multiple comparisons tests between individual groups with Prism GraphPad (version 8.2.1). No significant differences in the expression levels of *BCL2L1* were detected between control, 16pdel or 16pdup DA neurons. Adjusted p-values: control vs 16pdel p=0.93, control vs 16pdup p=0.42, 16pdel vs 16pdup p=0.55. Source data are provided as a Source Data file.

Supplementary Table 2. Primer sequences of the forward and reverse primers that were used in qRT-PCR experiments.

**Gene Name**	**Forward Primer 5'-3'**	**Reverse Primer 5'-3'**
OCT4	GAG GCA ACC TGG AGA ATT TG	CAC TCG GAC CAC ATC CTT CT
NANOG	TCT TCC TAC CAC CAG GGA TG	ACT GGA TGT TCT GGG TCT GG
FOXA2	TTT AAA CTG CCA TGC ACT CG	CCT CGG GCT CTG CAT AGT AG
EN1	GAG CGC AGG GCA CCA AA TA	CGA GTC AGT TTT GAC CAC GG
TH	CCG TGC TAA ACC TGC TCT TC	ATG GTG GAT TTT GGC TTC AA
LRRC4C	ACG GAA TGC CTT TGA CAA CC	TTT ATC CAC CAG CTG AGC CA
G0S2	AGG AGA TGA TGG CCC AGA AG	TGC ACA CAG TCT CCA TCA GG
TFRC	AGA TTC CTG GTT CGG GTG TT	TGA AGA GAC TCA CTG CTG CA
GRIK3	AAG GAG CTC ATC GAC CAC AA	AGT GTC ATG AAG GGC TTG GA
EPHA7	GGG AAC GGA CCT ACT CAA CA	AAC CAG CAG CAG TAA AAG CC
WNT3	GCC CCA CTC GGA TAC TTC TT	AGG AAT ACT GTG GCC CAA CA
SH3GL2	GAT GTT ATG CTG GCT GGC TC	GCC CAC AAG TCA ACC AAA GT
DRD2	GCT GGA GAT GGA GAT GCT CT	GCA TGC CCA TTC TTC TCT GG
SLC6A2	TGC CTT CTT GAT CCC GTA CA	GGG CAG ATT TTC CAA ACG GT
PCDHB5	CCA GAG CTA CCA CTA CGA GG	CTA TTT CTT CAC CAG CGC CC
GRIA4	ACA CAC ACA CAA GTC CCT CA	CAT GTG CTG CTT TGG AGG TT
DSCAM	AGG GCT GCA GGA TCA GTA GA	TCG TAA GTG GAG GAG GCA CT

SYN3	TGA GCA GAT CCT TCA AGC CA	TGA GCT GAG AGA ACA CCC AG
KCND2	GTG ACT TTA CCA GGA GCC CT	CGC CGC CAT GAT TAC TTG AA
GSG1L	GTC GCC CAC ATG ATG TAC AC	TTG GTG TAG GAG TTG AGC GT
OPRM1	GAC TGA TGA TCT TGC GCC TC	CAG ACG ATG AAC ACA GCC AC
KCTD13	ATC GTC CAT GCC AAA GTG TG	AAG GAG GTA GGG AGG ATG GT
CORO1A	CTC CTC TTC CTC CTC CTC CT	CGT ATG TGA AGA GCC CCA GT
TMEM219	AGG AAC AAG ACC CGG ACA TT	GTC CTT TCT GTG GTC ACC CT
TAOK2	AAG GAG GTG CGG TTC TTA CA	ACA GCT GCG ATC TCT ACC TC
CDIPT	CCT TGC TGA AGT CGC TCA TC	CCA GCG TCA CTT CTT CTT GG
MAPK3	CTC CCG CCA GAC TGT TAG AA	TCA GCA AAG GAG AGA GGT GG
DOC2A	TCT GAA GGA GTT GGA GCA GG	GAC GTA GGG GTC CGA GTA AC
PPP4C	TGG TAG AGG AGA GCA ACG TG	TCG CCA CCT ACT CTG AAC AG
GAPDH	CAG CCT CAA GAT CAT CAG CA	TGT GGT CAT GAG TCC TTC CA
QPRT	CTA AAC CGG AAG AGG ATG ACAC	CAT TGG CCA CTG ACC CTA AA
BOLA2	TTC CTA ACC CCA TCT CTC GC	TAA TGG CTG TGC AGA TCC CA
KIF22	TGG AAG GCA GTG ATG TGG AT	AGT GGT GTT CGA TTC TGG GT
SLX1A	TGG GGT TCA CTG TCA ACA CT	GAC GAG CAC CAT CTC CCA G
SULT1A4	GCA ACG CAA AGG ATG TGG CA	TCC GTA GGA CAC TTC TCC GA
MAZ	CAA CAG AAC GGC CCT TCA AA	CAT GGC ACT TTC TCC TCG TG
ASPHD1	CAG GAA GGA GCG AGT AGG AG	CTT CTT TCT CTC CGG CCT CA
INO80E	TTC CCT TCT GAC TAC CTG GC	CAC CGC CAT CTT GAG TTT CC

SEZ6L2	CTC TAC TGT TCC CTT GGC CA	GCT CTC CCC TTT GTC CTT CT
HIRIP3	GAA GAG CAG AAA GAG GCA GC	ACC TCT TCC CAC CCT TAA GC
FAM57B	AGT TTC ATC GGA CTC CCT GG	GTT AGG GGT GGA GAA GGG AC
GDPD3	GCT TGG TGA GAC GCT ATG AC	TAG GAA AGC AGC ACC CAG AA
C16orf54	CAG GCT ATG TGG ACT AGG GG	GCG ATC TTG AAG GCT TCT GG
PRRT2	CTC CCT CCC TAG CTG ACT TG	AGA TGG GAG AGG GGA GAG AG
PAGR1	AGC TGT TAC TTA GGG GCG TT	TCT TAA CCC CAC TCC TGC TG
MVP	AGA TCA CCA CCA ACT CCC AG	AGC TCC AAA AGT TCC TTG CG
FIMP	CTG TCC CCT CTG TTT CCC AT	CCT GGG AGG TGC TTT ACA GA
ALDOA	CGC GTT CTC TCC TTG AAT CC	TCA TGT TGA AGC TGG ACC CT
TBX6	GCC CCT TCC CTC TAC CAT AC	AGG TTT GTG ATG GAG GCA GA
YPEL3	TAT GGC CTG TGT TCT GCT CT	CAA ACG CTA CAG AAC GAG GG

Supplementary Table 3. Primary antibodies used in immunocytochemical analyses and western blot experiments

Antibody	Vendor	Catalog nr	Host	Dilution
Anti-Tyrosine hydroxylase	Pel-Freeze	P40101	Rabbit	1:600
Anti-Synapsin 1	EMD Millipore	AB1543P	Rabbit	1:500
Anti-PSD95	Neuro Mab	75-028	Mouse	1:80
Anti-TUJ1/b-III-Tub	EMD Millipore	AB9354	Chicken	1:200
Anti-TUJ1/b-III-Tub	Biolegend	801201	Mouse	1:600
Anti-Synaptophysin	Abcam	ab8049	Mouse	1:80
Anti-DAT	EMD Millipore	MAB369	Rat	1:100
Anti-Ki67	Abcam	ab15580	Rabbit	1:500
Anti-FOXA2/HFNb3	Santa-Cruz	Sc-374376	Mouse	1:50
Anti-RHOA	Cell Signaling	2117S	Rabbit	1:1000
Anti-NURR1	Thermo Fisher Scientific	MA1195	Mouse	1:250
Anti-LMX1A	Abcam	Ab139726	Rabbit	1:50
Anti-KCTD13	Invitrogen	PA5-60403	Rabbit	1:250
Anti-OCT4	Thermo Fisher Scientific	701756	Rabbit	1:250
Anti-NANOG	Thermo Fisher Scientific	PA1097	Rabbit	1:200
Anti-Tra1-60	Invitrogen	MA1-023	Mouse	1:150
Anti-CTIP2	Abcam	Ab18465	Rat	1:400
Anti-HB9	Thermo Fisher Scientific	PA5-67195	Rabbit	1:400
Anti-GABA	Sigma	A2052	Rabbit	1:1000
Anti-SOX2	ED Millipore	AB5603	Rabbit	1:500
Anti-Nestin	R&D Systems	MAB1259	Mouse	1:50
Anti-GFAP	Thermo Fisher Scientific	OPA106100	Rabbit	!:200
Anti-Cullin3	Thermo Fisher Scientific	PA517397	Rabbit	1:1000
Anti-LDHA	Cell Signaling	3582S	Rabbit	1:1000
Cofilin	Abcam	Ab54532	Mouse	1:1000

Phospho-Cofilin	Cell Signalling	5175S	Rabbit	1:1000
Total S6	Santa-Cruz Biotechnology	Sc-74459	Mouse	1:1000
Phospho-S6 (S240/244)	Cell Signalling	5364/L	Rabbit	1:1000
NCAM (CD56)-APC	Novus Biologicals	NBP2-34397APC	Mouse	1:100

Supplementary Table 4. Secondary antibodies used in immunocytochemical analyses and western blot experiments.

Antibody	Vendor	Catalog nr	Host	Dilution
Anti-mouse Alexa 488	Invitrogen/ThermoFisher Scientific	A11001	Goat	1:400
Anti-mouse Alexa 594	Invitrogen/ThermoFisher Scientific	A11004	Goat	1:400
Anti-chicken Alexa 647	Invitrogen/ThermoFisher Scientific	A21449	Goat	1:400
Anti-rabbit Alexa-488	Invitrogen/ThermoFisher Scientific	A11008	Goat	1:400
Anti-rabbit Alexa 568	Invitrogen/ThermoFisher Scientific	A11011	Goat	1:400
Anti-mouse 680RD	LI-COR Biotech.	926-68022	Donkey	1:10,000
Anti-mouse 800CW	LI-COR Biotech.	P/N 925-32210	Goat	1:10,000
Anti-rabbit 680RD	LI-COR Biotech.	P/N 925-68071	Goat	1:10,000
Anti-rabbit 800CW	LI-COR Biotech.	P/N 925-32211	Goat	1:10,000

Supplementary Table 5. Disease-relevant findings in the patients with 16p11.2 CNV syndromes, in the mouse models of 16p11.2 del/dup, and in the iPSC-derived neuronal cells with 16p11.2 CNVs.

**CNV**	**16p11.2 Del**	**16p11.2 Dup**	**References**
**Clinical phenotypes of the patients; Physiological phenotypes**	-Increased BMI and macrocephaly.<br>-increased white matter volume in children and adult patients.<br>-16pdel patients had increased total intracranial volume, total grey matter volume, and cortical surface compared to 16pdup patients.<br>-Cortical thickness was decreased compared to control.	-Decreased BMI and microcephaly.<br>- decreased supratentorial white matter volume in children and adult patients.<br>-16pdup patients had decreased total intracranial volume and cortical surface compared to control and 16pdel patients.<br>-total grey matter volume was decreased compared to 16pdel patients.<br>-Cortical thickness was decreased compared to control.	*Bochukova et al., 2010; Jacquemont S, et al., 2011; Maillard, et al., 2015; Shinawi M, et al., 2010.*
**Clinical phenotypes of the patients; Behavioral and psychological phenotypes**	-ADHD<br>-Anxiety<br>-Articulation disorder<br>-Behavioral disorder<br>-ASD<br>-Coordination disorder<br>-Enuresis disorder<br>-Language disorder<br>-Mood disorder<br>-Intellectual disability<br>-Stereotyped motor disorder<br>-seizures	-ADHD<br>-Anxiety<br>-Articulation disorder<br>-Behavioral disorder<br>-ASD<br>-Coordination disorder<br>-Enuresis disorder<br>-Language disorder<br>-Mood disorder<br>-Intellectual disability<br>-schizophrenia<br>-bipolar disorder<br>-depression<br>-seizures	*Giaroli,et al., 2014; Green, et al., 2016; Hanson,et al., 2015; Maillard, et al., 2015; McCarthy, et al., 2009; Niarchou, et al., 2019; Rees et al., 2014; Stefansson, et al., 2014; Shinawi, 2010; Walsh, 2011.*
**Mouse brain development**	-Increased volume of basal ganglia, dorsal striatum,<br>-Increased volume of nucleous accumbens and globus pallidus.<br>-Increased thickness of motor cortices.<br>-Reduced thickness of auditory cortex, and insular cortices,<br>-Mesodiencephalic structures; thalamus, hypothalamus and superior and inferior colliculi, were increased.<br>- Increased volume of basal	-No significant differences were detected between 16pdup brain regions vs control mice brain regions.<br>- Decreased volume of basal forebrain, superior colliculus, fornix, hypothalamus, mammillothalamic tract, medial septum, midbrain, and periaqueductal compared to 16pdel mice.	*Horev et al., 2011; Portmann, et al., 2014.*

	forebrain, superior colliculus, fornix, hypothalamus, mammillothalamic tract, medial septum, midbrain, and periaqueductal.		
**Mouse behavioral phenotypes**	- Half of the 16p11.2 deletion mice die postnatally.<br>-Difficult to adapt changes.<br>-Sleeping abnormalities.<br>-Repetitive and restricted behaviors.<br>-16pdel mice had similar phenotypes than rats with hypothalamic or nigrostriatal lesions associated to Parkinson's disease.<br>-Hyperactivity<br>-Circling<br>-Deficits in movement control.	-Reduced activity during dark cycle.<br>-No changes in activity during light cycle compared to control animals.<br>-No difficulties to adapt changes.	*Horev et al., 2011; Portmann, et al., 2014.*
**Phenotypes of mouse neural progenitors and neurons**	-In the striatum the 16pdel mice have increased number of Drd2+ MSNs.<br>-Reduced cortical Drd1+ neurons.<br>-Significant decrease in paired-pulse ratio in MSNs.<br>-Increased mEPSCs and sEPSCs frequency in MSNs.	- 16pdup pyramidal neurons exhibit a significant increase in dendritic complexity compared with control neurons.<br>- *MAPK3*/ERK1 plays important role in the gene hub related to the 16p11.2 network and broader schizophrenia-associated CNV gene networks.<br>- Treatment with ERK inhibitor reduced dendritic complexity over whole dendritic content in the 16pdup cells, compared with vehicle treated neurons.	*Blizinsky, et al 2016; Portmann, et al., 2014.*
**Phenotypes of human iPSC-derived neural cells and neurons**	-Increased soma of TH-positive DA neurons with 16pdel.<br>-Increased early neuronal differentiation and increased expression of neuronal genes in DA neuron population with 16pdel.<br>-Increased PSD95/SYN1 puncta and synaptophysin puncta on DA neurons.<br>-Reduced KCTD13 expression that leads to increased RHOA expression in the DA neurons.<br>-Increased phosphorylation of Cofilin in DA neurons rescued by Rhosin treatment.<br>- Cortical neurons with 16pdel had increased soma size.<br>-Cortical neurons with 16pdel had decreased density of excitatory synapses	-No significant change in the DA neuron soma size with 16pdup.<br>-Downregulation of neuronal genes in DA neuron population.<br>-Increased number of primary neurites in DA neurons with 16pdup.<br>-Decreased synaptophysin puncta on DA neurons.<br>- Cortical neurons with 16pdup neurons had reduced soma size and dendrite length.<br>-Cortical neurons with 16pdup had decreased density of excitatory synapses VGLUT2/PSD95 and SYN1/HOM1.	*Deshpande et al., 2017; Sundberg, et al. (current study).*

	VGLUT2/PSD95 and SYN1/HOM1.		
**Functionality of the human iPSC-derived neurons**	-DA neuron networks display hyperactivity and increased synchronization.<br>-DA neuron networks display increased bursting.<br>-DA neurons were hyperexcitability and had increased sEPSCs.<br>-Rhosin treatment rescued network hyperactivity in 16pdel DA neurons.<br>-Cortical neurons with 16pdel display hypoexcitability due to increased soma size.<br>-The amplitude of mEPSCs was significantly increased in 16pdel cortical neurons.<br>-No differences in the frequency of mEPSCs were detected between control and 16pdel cortical cells.	-DA neurons with 16pdup did not display significant deficits in neuronal excitability or network function.<br>-Cortical neurons with 16pdup did not display significant deficits on neuronal excitability.<br>-The amplitude of mEPSCs was significantly increased in 16dup cortical neurons.<br>-No differences in the frequency of mEPSCs were detected between control and 16pdup cortical cells.	*Deshpande et al., 2017; Sundberg, et al. (current study).*
