# Supplementary material for: 16p11.2 deletion is associated with hyperactivation of human iPSC-derived dopaminergic neuron networks and is rescued by RHOA inhibition in vitro
Source: Nat Commun. 2021 May 18;12:2897. doi: 10.1038/s41467-021-23113-z (PMC8131375; doi:10.1038/s41467-021-23113-z)
Supplement: Supplementary file 3 — Description of Additional Supplementary Files [file 41467_2021_23113_MOESM3_ESM.docx]

Description of Additional Supplementary Files

Title: Supplementary Data 1.

Description: Gene ontology categories of Module 19 are presented. Categories are presented with smallest -Log10 p-value 4.43 until highest -Log10 p-value 1.00 (Sheet 1). List of genes included in the M19 (Sheet 2). The RNAseq gene expression data of control cells vs 16pdup cells is presented (Sheet 3). The statistical analyses was done with modified Fisher’s Exact Test p-value, one sided, and correction for multiple comparisons was made with the FDR (Sheets 1, 3).

Title: Supplementary Data 2.

Description: Gene ontology categories in Module 25 are presented. Categories are presented with smallest -Log10 p-value 24.63 until highest -Log10 p-value 1.00 (Sheet 1). List of genes included in the M25 (Sheet 2). The RNAseq gene expression data of control cells vs 16pdel cells is presented (Sheet 3). The statistical analyses was done with modified Fisher’s Exact Test p-value, one sided, and correction for multiple comparisons was made with the FDR (Sheets 1, 3).

Title: Supplementary Data 3.

Description: IPA analyses of the disease categories (Sheet 1) and upstream regulators of the genes expressed in the M19 (Sheet 2) are presented. Statistical analyses was done with Right-Tailed Fisher’s Exact Test p-value, one sided. Data was not corrected for multiple comparisons.

Title: Supplementary Data 4.

Description: IPA analyses of the disease categories (Sheet 1) and upstream regulators of the genes expressed in the M25 (Sheet 2) are presented. Statistical analyses was done with Right-Tailed Fisher’s Exact Test p-value, one sided. Data was not corrected for multiple comparisons.
