# Supplementary material for: 16p11.2 deletion is associated with hyperactivation of human iPSC-derived dopaminergic neuron networks and is rescued by RHOA inhibition in vitro
Source: Nat Commun. 2021 May 18;12:2897. doi: 10.1038/s41467-021-23113-z (PMC8131375; doi:10.1038/s41467-021-23113-z)
Supplement: Supplementary file 8 — Reporting Summary [file 41467_2021_23113_MOESM8_ESM.pdf]

nature research

Corresponding author(s): Dr. Mustafa Sahin

Last updated by author(s): 2021/03/08

Reporting Summary

Nature Research wishes to improve the reproducibility of the work that we publish. This form provides structure for consistency and transparency in reporting. For further information on Nature Research policies, see our Editorial Policies and the Editorial Policy Checklist.

Statistics

For all statistical analyses, confirm that the following items are present in the figure legend, table legend, main text, or Methods section.

n/a	Confirmed	
☐	☒	The exact sample size (n) for each experimental group/condition, given as a discrete number and unit of measurement
☒	☐	A statement on whether measurements were taken from distinct samples or whether the same sample was measured repeatedly
☐	☒	The statistical test(s) used AND whether they are one- or two-sided<br>*Only common tests should be described solely by name; describe more complex techniques in the Methods section.*
☒	☐	A description of all covariates tested
☐	☒	A description of any assumptions or corrections, such as tests of normality and adjustment for multiple comparisons
☐	☒	A full description of the statistical parameters including central tendency (e.g. means) or other basic estimates (e.g. regression coefficient) AND variation (e.g. standard deviation) or associated estimates of uncertainty (e.g. confidence intervals)
☐	☒	For null hypothesis testing, the test statistic (e.g. F, t, r) with confidence intervals, effect sizes, degrees of freedom and P value noted<br>*Give P values as exact values whenever suitable.*
☒	☐	For Bayesian analysis, information on the choice of priors and Markov chain Monte Carlo settings
☒	☐	For hierarchical and complex designs, identification of the appropriate level for tests and full reporting of outcomes
☒	☐	Estimates of effect sizes (e.g. Cohen's d, Pearson's r), indicating how they were calculated

Our web collection on statistics for biologists contains articles on many of the points above.

Software and code

Policy information about availability of computer code

Data collection	MaxLab Live Software v. 20.1.6 (MaxWell Biosystems AG, Switzerland) was used for collection of the HD-MEA data in Maxwell Biosystems CMOS-HD-MEA system (MaxOne System, Maxwell Biosystems AG, Switzerland).<br>Maestro MEA and Axion Integrated Studio software AxIS v.2.5.1 (Axion BioSystems) was used for collection of LD-MEA data from Axion-MEA platform (Axion BioSystems, Atlanta, GA).<br>SutterPatch IPA v2.1.0 (Sutter Instruments, Sunnyvale, CA) was used for patch clamp recordings (https://www.sutter.com/AMPLIFIERS/sutterpatch_frame.html).<br>Molecular Devices software MetaXpress v.6.6.2.46 was used for imaging immunostained cells in ImageXpress MicroXLS Widefield High-Content Molecular Device microscope.<br>Zen-Blue software v. 8.1 (Zeiss) was used for imaging immunostained cells with LSM700 confocal microscope (Carl Zeiss, German).<br>Applied Biosystems Quant Studio Design and Analyses software v.1.5.1 (Thermo Fisher Scientific) was used for qRT-PCR.<br>Flow cytometry data was collected with FACSDiva software v.8.0.3 (BD Biosciences).
Data analysis	For the RNA seq analyses the initial quality control for the sequencing data was done with fastQC v.0.11.5. Reads were then mapped with STAR v.2.7 using an index created from release 91 of the HG38 build of the genome from Ensembl with associated transcript annotations. Cufflinks v.2.2.1 was used to create a merged transcriptome assembly from all samples, and this reference was used to quantify gene abundance from all samples. We performed differential expression analysis with LIMMA (v.3.46.0). We did Weighted Gene Co-expression Network Analysis using the WGCNA package (v.1.70-3) in R (v.4.0.3). Gene ontology analysis was performed using DAVID v.6.8 (https://david.ncifcrf.gov/). Ingenuity Pathway Analysis (IPA) (version 57662101) was used for pathway enrichment analysis in Ingenuity knowledge base (www.qiagen.com/ingenuity).<br>SutterPatch IPA v2.1.0 (Sutter Instruments, Sunnyvale, CA) was used for patch clamp data analyses (https://www.sutter.com/AMPLIFIERS/sutterpatch_f rame.html).<br>LD-MEA data was analyzed using Axion Integrated Studio software AxIS v.2.5.1 and the AxIs Metric Plotting Tool v. 1.5.11, (Axion BioSystems, Atlanta, GA). These summary files were collated and analyzed using custom R scripts (v.4.0.3).

The HD-MEA data was analyzed with Matlab (R2020b), and the code that were generated for HD-MEA data analyses has been downloaded to Github. The HD-MEA burst data was analyzed with costum code developed for burst analyses, published: Kapucu FE, Tanskanen JM, Mikkonen JE, Yla-Outinen L, Narkilahti S, Hyttinen JA. Burst analysis tool for developing neuronal networks exhibiting highly varying action potential dynamics. Front Comput Neurosci 6, 38 (2012).

Molecular Devices MetaXpress software v6.5.2.351 was used for image analyses of the ImageXpress MicroXLS Widefield High-Content Molecular Device microscope images. ImageJ/(Fiji version 1.0) was used for soma size analyses.

For statistical analyses the GraphPad Prism version 8.2.1. was used.

Flow cytometry data was analyzed with FloJo v9.9.6 (BD Biosciences).

For manuscripts utilizing custom algorithms or software that are central to the research but not yet described in published literature, software must be made available to editors and reviewers. We strongly encourage code deposition in a community repository (e.g. GitHub). See the Nature Research guidelines for submitting code & software for further information.

Data

Policy information about availability of data

All manuscripts must include a data availability statement. This statement should provide the following information, where applicable:

- Accession codes, unique identifiers, or web links for publicly available datasets
- A list of figures that have associated raw data
- A description of any restrictions on data availability

Data availability: The transcriptional RNAseq data has been deposited to the European Genome-phenome Archive (EGA) under the study accession number: EGAS00001005137, and the data accession number: EGAD00001007072, https://urldefense.com/v3/__https://ega-archive.org/studies/EGAS00001005137__;!!NZvER7FxgEiBAiR_!9rGbdjnqclX2ruflvu4FYl43k44Gy89MQGBXvq8Ug-1MZkvSBOI83KLYNVTpeKTpRjAN9AK1-jAhag$ and https://urldefense.com/v3/__https://ega-archive.org/datasets/EGAD00001007072__;!!NZvER7FxgEiBAiR_!9rGbdjnqclX2ruflvu4FYl43k44Gy89MQGBXvq8Ug-1MZkvSBOI83KLYNVTpeKTpRjAN9ALEJBXdQg$ and https://urldefense.com/v3/__https://ega-archive.org/dacs/EGAC00001002030__;!!NZvER7FxgEiBAiR_!9rGbdjnqclX2ruflvu4FYl43k44Gy89MQGBXvq8Ug-1MZkvSBOI83KLYNVTpeKTpRjAN9AJ2_xpo2A$. Other data are available in the Supplementary materials of this paper, and upon request from the corresponding author. Source data are provided with this paper in a Source Data file.

Field-specific reporting

Please select the one below that is the best fit for your research. If you are not sure, read the appropriate sections before making your selection.

☒ Life sciences ☐ Behavioural & social sciences ☐ Ecological, evolutionary & environmental sciences

For a reference copy of the document with all sections, see nature.com/documents/nr-reporting-summary-flat.pdf

Life sciences study design

All studies must disclose on these points

Sample size for each experiment was chosen based on previously reported studies and reproducibility of the data. Experiments were performed with human iPSC lines 3 clones/genotype, and the sample collections were done from at least 2-3 independent differentiation batches/cell line for each experiment, consisting at least 3 technical replicates when applicable.
The LD-MEA data that was generated from 6-16 well replicates/genotype (from two independent differentiation batches). Sample sizes were chosen based on previously published papers with similar study designs and statistical analyses (Sundberg M., et al 2018, Molecular Psychiatry, Winden K., et al 2019, Journal of Neuroscience).

Data exclusions

In the analyses of the sEPSCs, and soma area; the outliers were removed from the dataset with Prism-statistical program ROUT Q=1%. No data were excluded in the other data analyses.

Replication

All attempts for data replication were successful. Data replication were verified by analyzing the data from several technical and biological replicates of each sample per experiment. The results are presented as average values of replicates per sample and standard error of mean or standard deviation were calculated to present the variability between different replicates, n-values for each experimental group are outlined in the figure legends and materials and methods section.

Randomization

We were specifically comparing different cell types to control cells and comparing treatment vs non-treatment groups, that is why randomization of samples for current study was not applicable.
Each experimental group contained samples from all the three genotypes studied: control cell population, 16pdel population, and 16pdup population, and samples were always compared to the control cell line within the experimental group.

Blinding

Investigators were not blinded for all the experimental groups during the data collection and analyses, which is because the experiments were done with small sample groups and the mutant samples were always compared to the control samples. In these analyses a specific value were calculated for the control samples and the other samples were compared to that (eg qRT-PCR analyses, western blot analyses). The sample processing for the RNA-sequencing run and for the FACS-sorting were done by the Core-personnel who were blinded for the sample identity and for the experimental design.

Reporting for specific materials, systems and methods

We require information from authors about some types of materials, experimental systems and methods used in many studies. Here, indicate whether each material, system or method listed is relevant to your study. If you are not sure if a list item applies to your research, read the appropriate section before selecting a response.

Materials & experimental systems

n/a	Involved in the study
☐	☒ Antibodies
☐	☒ Eukaryotic cell lines
☒	☐ Palaeontology and archaeology
☒	☐ Animals and other organisms
☒	☐ Human research participants
☒	☐ Clinical data
☒	☐ Dual use research of concern

Methods

n/a	Involved in the study
☒	☐ ChIP-seq
☐	☒ Flow cytometry
☒	☐ MRI-based neuroimaging

Antibodies

Antibodies used

Anti-Tyrosine hydroxylase (Pel-Freeze, P40101); Anti-Synapsin 1 (EMD Millipore, AB1543P); Anti-PSD95 (Neuro Mab, 75-028); Anti-TUJ1/b-III-Tub (EMD Millipore, AB9354); Anti-TUJ1/b-III-Tub (Biolegend, 801201); Anti-Synaptophysin (Abcam, ab8049); Anti-DAT (EMD Millipore, MAB369); Anti-Ki67 (Abcam, ab15580); Anti-FOXA2/HFNb3 (Santa-Cruz, Sc-374376); Anti-RHOA (Cell Signaling, 2117S); Anti-KCTD13 (Invitrogen, PA5-60403); Anti-OCT4 (Thermo Fisher Scientific, 701756); Anti-NANOG (Thermo Fisher Scientific, PA1097); Anti-Tra1-60 (Invitrogen, MA1-023); Anti-CTIP2 (Abcam, Ab18465); Anti-HB9 (Thermo Fisher Scientific, PA5-67195); Anti-GABA (Sigma, A2052); Anti-SOX2 (ED Millipore, AB5603); Anti-Nestin (R&D Systems, MAB1259); Anti-GFAP (Thermo Fisher Scientific, OPA106100); Anti-Cullin3 (Thermo Fisher Scientific, PA517397); Anti-LDHA (Cell Signaling, 3582S); Cofilin (Abcam, Ab54532); Phospho-Cofilin (Cell Signalling, 5175S); Total S6 (Santa-Cruz Biotechnology, Sc-74459); Phospho-S6 (S240/244) (Cell Signalling, 5364/ L), Anti-NURR1 (Thermo Fisher Scientific, MA1195); Anti-LMX1A (Abcam, Ab139726); Anti-mouse Alexa 488 (Invitrogen/ ThermoFisher Scientific, A11001); Anti-mouse Alexa 594 (Invitrogen/ThermoFisher Scientific, A11004); Anti-chicken Alexa 647 (Invitrogen/ThermoFisher Scientific, A21449); Anti-rabbit Alexa-488 (Invitrogen/ThermoFisher Scientific, A11008), Anti-rabbit Alexa 568 (Invitrogen/ThermoFisher Scientific, A11011); Anti-mouse 680RD (LI-COR Biotech., 926-68022); Anti-mouse 800CW (LI-COR Biotech., P/N 925-32210); Anti-rabbit 680RD (LI-COR Biotech., P/N 925-68071); Anti-rabbit 800CW (LI-COR Biotech., P/N 925-32211); anti-NCAM(CD56)-APC (SPM128, Novus Biologicals, NBP2-34397APC); anti-mouse IgG1-APC (11711, Novus Biologicals, IC002A).

Validation

Validation of each antibody specificity and localization are presented in the manufacturers website and it includes lists of previous publications. For each antibody, we verified that the expression responded the specifications presented in the manufacturers website.

Anti-Tyrosine hydroxylase (Pel-Freeze, P40101); Species Reactivity: All mammalian and at least some non-mammalian forms of the enzyme in Western blots and in IHC/IF (https://www.pelfreez-bio.com/wp-content/uploads/2014/07/74075-PDS-P40101-Tyrosine-Hydroxylase-Antibody-Rabbit-Rev-02.pdf).
Anti-Synapsin 1 (EMD Millipore, AB1543P); Species Reactivity: bovine, human, mouse, rat. https://www.emdmillipore.com/US/en/product/Anti-Synapsin-I-Antibody,MM_NF-AB1543P?ReferrerURL=https%3A%2F%2Fwww.google.com%2F.
Anti-PSD95 (Neuro Mab, 75-028); Species Reactivity:Human, mouse, rat. https://www.antibodiesinc.com/products/psd-95-k28-43?variant=12783173730363.
TUJ1/b-III-Tub (EMD Millipore, AB9354); Species Reactivity:Human, mouse, rat. https://www.emdmillipore.com/US/en/product/Anti-Beta-III-Tubulin-Antibody,MM_NF-AB9354.
Anti-TUJ1/b-III-Tub (Biolegend, 801201);Species Reactivity:Human, mouse, rat. https://www.biolegend.com/en-us/products/purified-anti-tubulin-beta-3-tubb3-antibody-11580.
Anti-Synaptophysin (Abcam, ab8049); Species Reactivity: Mouse, Rat, Hamster, Cow, Human. https://www.abcam.com/synaptophysin-antibody-sy38-ab8049.html.Anti-DAT (EMD Millipore, MAB369); Species Reactivity: Human, mouse, monkey, rat. https://www.emdmillipore.com/US/en/product/Anti-Dopamine-Transporter-Antibody-NT-clone-DAT-Nt,MM_NF-MAB369.
Anti-Ki67 (Abcam, ab15580); Species Reactivity: Mouse, Rat, Sheep, Rabbit, Horse, Cow, Dog, Human, Pig, Indian muntjac, Monkey, Chinese hamster, Common marmoset, Syrian hamster. https://www.abcam.com/ki67-antibody-ab15580.html.
Anti-FOXA2/HFNb3 (Santa-Cruz, Sc-374376); Species Reactivity: mouse, rat and human. https://datasheets.scbt.com/sc-374376.pdf.
Anti-RHOA (Cell Signaling, 2117S); Species Reactivity: Human, mouse, rat, monkey, bovine. https://www.cellsignal.com/datasheet.jsp?productId=2117&images=1&protocol=0. Anti-KCTD13 (Invitrogen, PA5-60403); Species Reactivity: Human. https://www.thermofisher.com/order/genome-database/dataSheetPdf?producttype=antibody&productsubtype=antibody_primary&productId=PA5-60403&version=112.
Anti-OCT4 (Thermo Fisher Scientific, 701756); Species Reactivity: Human, mouse. https://www.thermofisher.com/order/genome-database/dataSheetPdfproducttype=antibody&productsubtype=antibody_ primary&productId=701756&version=112.
Anti-NANOG (Thermo Fisher Scientific, PA1097); Species Reactivity: Human. https://www.thermofisher.com/order/genome-database/dataSheetPdf?producttype=antibody&productsubtype=antibody_ primary&productId=PA1-097& version=112.
Anti-Tra1-60 (Invitrogen, MA1-023); Species Reactivity: Human. Antibody validation; https://www.thermofisher.com/order/genome-database/dataSheetPdf?producttype=antibody&productsubtype=antibody_primary&productId=MA1-023&version=112.
Anti-CTIP2 (Abcam, Ab18465); Species Reactivity:Human, mouse. https://www.abcam.com/ctip2-antibody-25b6-ab18465.html. Anti-HB9 (Thermo Fisher Scientific, PA5-67195); Species Reactivity:Human. https://www.thermofisher.com/antibody/product/HB9-Antibody-Polyclonal/PA5-67195. Anti-GABA (Sigma, A2052); Species Reactivity: wide range, rat, Drosophila. https://www.sigmaaldrich.com/catalog/product/sigma/a2052?lang=en&region=US.Anti-SOX2 (ED Millipore, AB5603); Species Reactivity:Human, mouse.https://www.emdmillipore.com/US/en/product/Anti-Sox2-Antibody,MM_NF-AB5603.
Anti-Nestin (R&D Systems, MAB1259); Species Reactivity:Human. https://www.rndsystems.com/products/human-nestin-antibody-196908_mab1259. Anti-GFAP (Thermo Fisher Scientific, OPA106100); Species Reactivity: Human, Mouse, Rat, Zebrafish. https://www.thermofisher.com/antibody/product/GFAP-Antibody-Polyclonal/OPA1-06100.
Anti-Cullin3 (Thermo Fisher Scientific, PA517397); Species Reactivity: Human, Mouse, Non-human primate, Rat. Antibody validation :https://www.thermofisher.com/antibody/product/Cullin-3-Antibody-Polyclonal/PA5-17397.
Anti-LDHA (Cell Signaling, 3582S); Species Reactivity: Human, monkey. https://www.cellsignal.com/products/primary-antibodies/

ldha-c4b5-rabbit-mab/3582.
Cofilin (Abcam, Ab54532); Species Reactivity:Mouse, Rat, Human. Antibody validation: https://www.abcam.com/cofilin-antibody-ab54532.html.
Phospho-Cofilin (Cell Signalling, 5175S); Species reactivity: Human, mouse, monkey, rat, bovine. https://www.cellsignal.com/ products/primary-antibodies/phospho-cofiln-ser3-77g2-rabbit-mab/3313?utm_strategy=lev&utm_conv=mon&utm_stage=ous&utm_tactic=ppc&utm_region=hq&gclsrc=aw.ds&&gclid=CjwKCAiAt9z-BRBCEiwA_bWv-OomlsRFCwn8fk-6zQANaJICFyGloNzlhcWdYB4cHL68xMvB7ykIexoCqxMQAvD_BwE.
Total S6 (Santa-Cruz Biotechnology, Sc-74459); Species reactivity; mouse, rat and human. https://www.scbt.com/p/ribosomal-protein-s6-antibody-c-8. Phospho-S6 (S240/244) (Cell Signalling, 5364/L); Species reactivity: Human, mouse, monkey, rat. https:// www.cellsignal.com/products/primary-antibodies/phospho-s6-ribosomal-protein-ser240-244-d68f8-xp-rabbit-mab/5364?utm_strategy=lev&utm_conv=mon&utm_stage=ous&utm_tactic=ppc&utm_region=hq&gclsrc=aw.ds&&gclid=CjwKCAiAt9z-BRBCEiwA_bWv-MAYaTnhiCBnSJVsQBoxqXOq24iDP6j79Py48txNHBqprv5dycRFHRoCrKwQAvD_BwE.
Anti-NURR1 (Thermo Fisher Scientific, MA1195); Species reactivity: Human, Mouse, Rat. https://www.thermofisher.com/antibody/product/Nurr1-Antibody-clone-N1404-Monoclonal/MA1-195.
Anti-LMX1A (Abcam, Ab139726); Species reactivity: Mouse, Rat, Human.https://www.abcam.com/lmx1a-antibody-c-terminal-ab139726.html.
Anti-NCAM(CD56)-APC (SPMl28, Novus Biologicals, NBP2-34397APC);Species reactivity:Human, rat, zebrafish. https://www.novusbio.com/products/ncam-1-cd56-antibody-spm128_nbp2-34397apc.

Eukaryotic cell lines

Policy information about cell lines

Cell line source(s)

Human iPSCs were derived from fibroblast line GM08330, purchased from Coriell Institute for Medical Research and previously described (Tai et al,Nat Neurosci. 2016 Mar;19(3):517-22. doi: 10.1038/nn.4235). CRISPR-Cas9 edited 16pll.2 deletion and 16pll.2 duplication iPSC lines were generated from the GM08330 iPSCs and characterized with RNAseq, aCGH and WB by Tai and colleagues (Tai et al 2016), and provided to this study by Dr. Tai and Dr. Gusella from Massachusetts General Hospital, Boston, MA, USA.

Authentication

CRISPR-Cas9 edited 16pll.2 deletion and 16pll.2 duplication lines were generated and characterized with RNAseq, aCGH and WB in Tai et al study (Nat Neurosci. 2016 Mar;19(3):517-22. doi: 10.1038/nn.4235.), and SNP array (Supplementary Table 1a) and karyotyping and qRT-PCR and immunocytochemistry (Supplementary Figure 1).

Mycoplasma contamination

Cells were tested negative for mycoplasma by PCR.

Commonly misidentified lines
(See ICLAC register)

No misidentified lines were used.

Flow Cytometry

Plots

Confirm that:

☒ The axis labels state the marker and fluorochrome used (e.g. CD4-FITC).

☒ The axis scales are clearly visible. Include numbers along axes only for bottom left plot of group (a 'group' is an analysis of identical markers).

☒ All plots are contour plots with outliers or pseudocolor plots.

☒ A numerical value for number of cells or percentage (with statistics) is provided.

Methodology

Sample preparation

Human iPSC-derived DA precursors were sorted with NCAMhigh+ selection using FACS-Aria II (BD Biosciences) after 27-32 days of differentiation in vitro. During sample preparation the adherently cultured neural cells were dissociated into single cells with Accutase (Innovative Cell Technologies) and DNAse 100 Units/ml 20 min at +37C. Cells were then washed and stained with NCAM-antibody (CD56-APC, Novus Biologicals) l:l00 dilution in 500ul suspcnsion, for 20 min at room temperature. Cells were washed twice and suspended into 2% BSA-HBSS-P/S and filtered through nylon mesh (35 uM, BD Falcon Cell Strainer cap). Cells were sorted with low sheath pressure 20 PSI, and with sterile PBS as fluidics, and 100 um nozzle. Sorted cells were collected into Neurobasal medium supplemented with B27 and 1% BSA, centrifuged and suspended on Neurobasal media with B27 (Life Technologies), BDNF 20ng/ml, GDNF 20ng/ml (Peprotech), DAPT 2.5 uM (Cayman Chemical), cAMP 500 mM (Sigma), TGFb 1ng/ml (Peprotech), and Ascorbic acid 200 uM (AA, Sigma), and cells were plated to PDL/laminin coated coverslips for further differentiation.

Instrument

FACSAria II (BD Biosciences)

Software

BD FacsDiva (v. 8.0.3. BD Biosciences)

Cell population abundance

Purity of the populations were determined by re-running a fraction of the sorted cells through the FACS-machine and visualization of the sorted cells inside the gate that was used for positive selection for sorting (P4-gate).

Gating strategy

Gating strategy for sorting of NCAMhigh+ DA neurons; First, we selected the population of interest and excluded the cell debris based on the cell size with SSC-A and FSC-A parameters (P1 gate). P2 gate; we excluded the doublets with FSC-H and FSC-A gating. P3 gate; we selected single cells and excluded the doublets with SSC-H and SSC-W gating. P4-gate; we selected

the cells with highest fluorescent intensity for NCAM(CD56)-APC+ for sorting in dot-plot of APC-A (y-axis) and FSC-A (x-axis). Gating strategy is shown in the supplementary fig. 2.

☒ Tick this box to confirm that a figure exemplifying the gating strategy is provided in the Supplementary Information.
